# A Diffuse Fine Papular and Pustular Rash in a Man With Advanced Human Immunodeficiency Virus and Diabetes

**DOI:** 10.1093/cid/cix710

**Published:** 2018-01-18

**Authors:** Charle Viljoen, Khanyisile Dladla, Innocent Francis, Helen Wainwright, Graeme Meintjes

**Affiliations:** 1 Department of Medicine, Groote Schuur Hospital and University of Cape Town, South Africa; 2 Division of Dermatology, Groote Schuur Hospital and University of Cape Town, South Africa; 3 Division of Anatomical Pathology, National Health Laboratory Service, Groote Schuur Hospital and University of Cape Town, South Africa

**(See pages 475–6 for Photo Quiz.)**

Diagnosis: Disseminated tuberculosis with skin involvement.

Bacterial blood and urine cultures were negative. The chest radiograph, which showed bilateral hilar lymphadenopathy and bilateral nodular pulmonary infiltrates, raised the suspicion of disseminated tuberculosis (Figure 1). The patient’s cough was unproductive and sputum could not be obtained for tuberculosis (TB) investigations. A skin biopsy was done before he was started on empiric antituberculous treatment. The skin biopsy showed subcorneal pustules extending through the epidermis into the superficial dermis, with acid-fast bacilli seen on Ziehl-Neelsen stain. There were no fungal elements seen (periodic acid–Schiff and Grocott stains were negative) and the Brown and Brenn stain for bacterial infection was also negative. Cultures of the skin were positive for *Mycobacterium tuberculosis* after 17 days of incubation, susceptible to rifampicin and isoniazid. The rash, cough, and constitutional symptoms resolved within a few days after starting the antituberculous treatment. Antiretroviral therapy was commenced at his local clinic, where the patient completed 6 months of TB treatment and continues follow-up.

**Figure 1.**
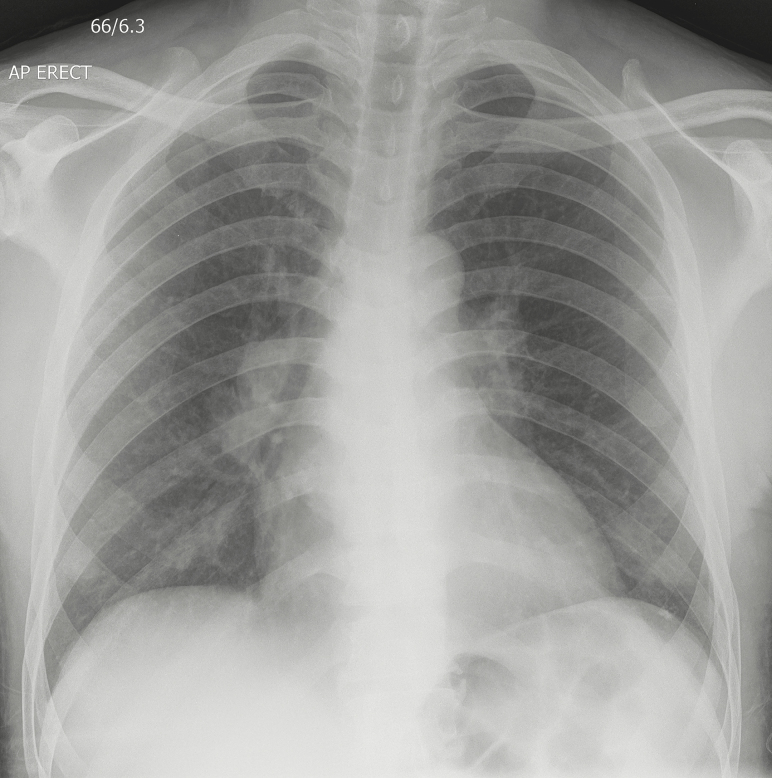
Chest radiograph with bilateral hilar lymphadenopathy and bilateral nodular pulmonary infiltrates.

Every year, 8–9 million new cases of TB are diagnosed [1]. The incidence of extrapulmonary TB has significantly increased in the last few decades with the increasing prevalence of human immunodeficiency virus (HIV), diabetes, cancer, and chronic kidney disease [1, 2]. However, cutaneous TB is rare, accounting for <2% of all TB cases [3–5]. Cutaneous TB has a diverse clinical presentation and is classified according to the mode of transmission: direct inoculation from an exogenous source (eg, tuberculous chancre and tuberculosis verrucosa cutis), or from an endogenous source, either spreading contiguously or by autoinoculation (eg, scrofuloderma and tuberculosis cutis orificialis), alternatively by hematogenous spread (eg, lupus vulgaris, miliary tuberculosis, tuberculous gumma) or presenting as tuberculids, which are hypersensitivity reactions to the TB bacillus (including erythema induratum, papulonecrotic tuberculid, and lichen scrofulosorum) [1]. Cutaneous miliary TB, as in this case, manifests with diffuse erythematous macules, papules, pustules, and papulovesicles. Miliary TB is an uncommon manifestation of tuberculous infection, which usually presents with pulmonary and meningeal involvement. Among disseminated TB cases, the skin is rarely involved [6]. The first report of cutaneous miliary TB in the setting of HIV was reported by [7], but has since only rarely been reported [8].

In the immunocompromised patient, the presentation of a similar rash and constitutional symptoms should prompt a differential diagnosis including bacterial infections of the skin (such as staphylococci, secondary syphilis), fungal infections (cryptococcosis, histoplasmosis, and emmonsiosis), and mycobacterial infections other than TB [9]. Clinicians should therefore have a low threshold to do a skin biopsy for specialized stains and culture to make an accurate diagnosis and direct appropriate treatment. The gold standard for diagnosing cutaneous TB is mycobacterial culture of skin biopsy followed by species identification [4, 5]. Standard pulmonary TB treatment regimens are sufficient to treat cutaneous TB [3, 5]. Delay in treatment adversely affects the prognosis [4].

## Note

***Potential conflicts of interest.*** All authors: No reported conflicts of interest. All authors have submitted the ICMJE Form for Disclosure of Potential Conflicts of Interest. Conflicts that the editors consider relevant to the content of the manuscript have been disclosed.
